# Electromagnetic Imaging of Anisotropic Objects Using a Self-Attention Perceptual Generative Adversarial Network

**DOI:** 10.3390/s26123705

**Published:** 2026-06-10

**Authors:** Po-Hsiang Chen, Chien-Ching Chiu, Yang-Han Lee, Eng Hock Lim

**Affiliations:** 1 Department of Electrical and Computer Engineering, Tamkang University, Tamsui 251301, Taiwan; phchen@mail.tku.edu.tw (P.-H.C.); yhleepp@gmail.com (Y.-H.L.); 2 Department of Electrical and Electronic, University Tunku Abdul Rahman, Kajang 43200, Malaysia; limeh@utar.edu.my

**Keywords:** electromagnetic inverse scattering, anisotropic media, deep learning, generative adversarial networks, self-attention

## Abstract

Reconstructing high-resolution images of anisotropic targets in microwave imaging remains a challenging problem due to the strong directionality of electromagnetic responses and the inherent nonlinearity of the inverse scattering process. To address these issues, we propose a novel Perceptual Generative Adversarial Network (PGAN) enhanced with a Self-Attention mechanism for anisotropic electromagnetic imaging. The perceptual loss encourages the preservation of high-level structural features, while the Self-Attention module enables the model to capture long-range dependencies and directional correlations that are critical in representing anisotropic material distributions. This joint architecture is trained to refine coarse permittivity estimates obtained from conventional Back-Propagation Schemes (BPSs). Numerical simulations and validation using measured experimental data demonstrate that the proposed method achieves improved reconstruction accuracy and structural similarity compared with the PGAN without SA and U-Net. In particular, PGAN with SA reduces the Root Mean Square Error (RMSE) by 15.1% and improves the Structural Similarity Index Measure (SSIM) by 3.8%, confirming its effectiveness in recovering fine-scale details and enhancing reconstruction quality. These results suggest that the proposed framework offers a promising solution for robust and high-resolution electromagnetic imaging in geophysical and remote sensing applications.

## 1. Introduction

Electromagnetic inverse scattering remains a fundamental yet inherently challenging problem in geophysical and remote sensing applications, including subsurface exploration, environmental monitoring, and nondestructive testing [1,2,3]. Despite extensive research efforts, reconstructing high-resolution images of anisotropic media continues to pose significant difficulties due to the intrinsic direction-dependent electromagnetic behavior—where material properties vary with incident wave orientation—and complex scattering mechanisms such as multiple scattering and strong nonlinear interactions [4,5]. Traditional inversion algorithms, including iterative Born approximations and regularization-based optimization techniques, have been widely studied and applied [6,7]. However, these methods often suffer from high computational costs, owing to repeated forward modeling and iterative updates, and are sensitive to noise, thereby limiting their effectiveness in real-time and practical imaging scenarios.

In recent years, the rapid advancement of artificial intelligence (AI) has profoundly reshaped many scientific and engineering domains, particularly in solving high-dimensional, ill-posed, and nonlinear inverse problems. Deep learning, as a leading approach within AI, has demonstrated exceptional capability in learning complex input–output mappings and extracting hierarchical representations from large-scale data using multilayer neural architectures. These advances have enabled significant progress in diverse fields such as computer vision, natural language processing, and medical imaging [8,9,10], and have recently begun to influence electromagnetic imaging. Within this context, deep learning provides a promising alternative to conventional model-driven solvers, enabling fast, noise-robust, and potentially more accurate reconstructions in complex environments.

To enhance electromagnetic image reconstruction, various learning-based methods have been proposed. In 2021, Guo introduced a novel GAN designed to enhance the resolution of preliminary reconstructions [11]. In 2022, Liu proposed a physics-inspired deep unrolling network, which integrates traditional model-based priors into a learnable architecture tailored for solving nonlinear inverse scattering problems. Numerical experiments demonstrated that physics-inspired deep unrolling network achieves superior accuracy while requiring fewer trainable parameters than conventional deep learning models, highlighting its potential for combining physical constraints with data-driven inference [12]. In the same year, Chiu investigated the reconstruction of uniaxial anisotropic objects using a two-stage approach. The method employed Transverse magnetic (TM) and Transverse electric (TE) wave excitations to illuminate the target, followed by an initial estimate using a Back-Propagation Scheme (BPS) and Dominant Current Scheme (DCS), and concluded with a CNN-based refinement for accurate image reconstruction [13]. In 2023, Zhang et al. developed an enhanced two-step deep learning framework to decouple frequency extrapolation and spatial reconstruction. The first network extrapolates high-frequency responses from limited low-frequency data, while a second network reconstructed the scatterer. This modular design improved reconstruction quality and generalization performance, especially under data scarcity, and outperformed conventional single-stage and model-based approaches [14]. In 2024, Yao presented a Conditional Deep Convolutional Generative Adversarial Network (CDCGAN) that directly reconstructs high-contrast scatterers from measured scattered fields. The framework jointly minimized the discrepancy between generated and true contrast images, as well as between generated and observed fields. Experimental results verified that CDCGAN was capable of accurately reconstructing strongly scattering objects under various noise levels [15].

More recently, attention mechanisms have been incorporated into electromagnetic imaging architectures to further improve reconstruction fidelity. In 2023, Xu proposed a learning-assisted inversion strategy combining Fourier basis expansion with a self-attention-equipped GAN. The initial permittivity estimate obtained from low-frequency measurements was refined through super-resolution reconstruction, with the attention mechanism capturing long-range dependencies and improving reconstruction accuracy. The approach demonstrated excellent generalization and stability, even under strong scattering conditions [16]. In 2024, Du introduced a globally perceptive architecture by integrating a global self-attention layer with convolutional operations. This configuration effectively captured both global and local dependencies, resulting in improved reconstruction performance across various scenarios [17]. In 2025, Maricar developed an Attention-gated U-Net (ATTN-Unet) architecture tailored for electromagnetic inverse scattering. The network incorporated attention gates to selectively enhance feature propagation and suppress irrelevant regions. Experimental results indicated that ATTN-Unet not only outperformed existing benchmarks in both image quality and computational efficiency, but also generalized well to real experimental data, emphasizing its potential for practical deployment in microwave imaging systems [18].

Although GAN-based refinement and attention mechanisms have been introduced in recent electromagnetic imaging studies, most existing approaches mainly focus on isotropic or scalar contrast reconstruction, super-resolution enhancement, or general feature extraction from scattered-field data. In contrast, the present work focuses on anisotropic electromagnetic inverse scattering, where the dielectric properties are direction-dependent and the TE-polarized case involves coupled transverse field components. The proposed method differs from previous GAN- or attention-based approaches by first using the anisotropic BPS formulation to obtain a physically meaningful initial reconstruction, which is then refined by a PGAN with embedded self-attention. In addition, the loss function combines pixel-wise reconstruction accuracy, perceptual/adversarial consistency, and structural similarity, thereby improving both quantitative accuracy and structural preservation of anisotropic dielectric profiles.

Motivated by the aforementioned developments, this work presents a novel deep learning framework tailored for electromagnetic inverse scattering of anisotropic media. Specifically, we propose a PGAN integrated with a self-attention mechanism to achieve high-resolution and high-fidelity image reconstruction from scattered-field measurements. Unlike conventional GAN-based models, the proposed PGAN incorporates a perceptual loss that enforces feature-level consistency between the reconstructed and ground-truth images by leveraging a pretrained feature extractor. This design enables the network to better preserve structural and textural details that are critical for interpreting complex scatterers. Furthermore, a self-attention module is embedded into the generator architecture to enhance the modeling of long-range spatial dependencies, allowing the network to effectively capture correlations between distant scattering features that are especially important in imaging anisotropic and strongly inhomogeneous objects. The discriminator is jointly optimized with the generator under an adversarial loss, guiding the reconstruction process towards producing visually realistic and physically plausible results. Our contributions are as follows:

1. We propose a novel PGAN with embedded self-attention mechanisms to address the anisotropic electromagnetic inverse scattering problem. The PGAN integrates a perceptual loss that preserves structural and semantic consistency between the reconstructed and ground-truth images, while the self-attention mechanism captures long-range spatial dependencies. Simulation results show that this design enhances the network’s ability to recover fine-scale features in anisotropic and strongly scattering media.
2. We further enhance the loss function by incorporating an SSIM-related term, which explicitly encourages the preservation of local structural information in the reconstructed images. By jointly optimizing pixel-wise fidelity, adversarial consistency, and structural similarity, the proposed objective function leads to improved reconstruction accuracy, particularly in preserving edges, textures, and fine-scale features of anisotropic scatterers.
3. We have successfully reconstructed anisotropic objects using the BPS, which is further extended by modifying the formulation to account for the inherent complexity of anisotropic media. In particular, TE-polarized waves, characterized by vector field components in both the x and y directions, pose greater challenges for image reconstruction compared to TM-polarized waves, which involve only scalar fields. The coupling between the x and y components in TE polarization leads to stronger multiple scattering effects and more intricate electromagnetic interactions, especially in anisotropic or uniaxial materials. As a result, the inverse scattering problem under TE polarization is significantly more ill-posed than its TM counterpart.
4. The feasibility and effectiveness of the proposed method are validated through extensive numerical experiments, demonstrating its improved reconstruction performance in reconstructing high-resolution images of anisotropic media under various scattering conditions.
5. Compared with the PGAN framework in [19], the proposed method further incorporates an SSIM-related structural loss and a self-attention mechanism to enhance boundary preservation, structural consistency, and long-range feature modeling in anisotropic electromagnetic imaging.

Section 2 presents the theoretical foundation of the study. Section 3 describes the proposed PGAN with self-attention (SA) framework. Section 4 provides the numerical results and related discussion. Finally, Section 5 concludes the paper.

## 2. Theory

**Figure 1:**
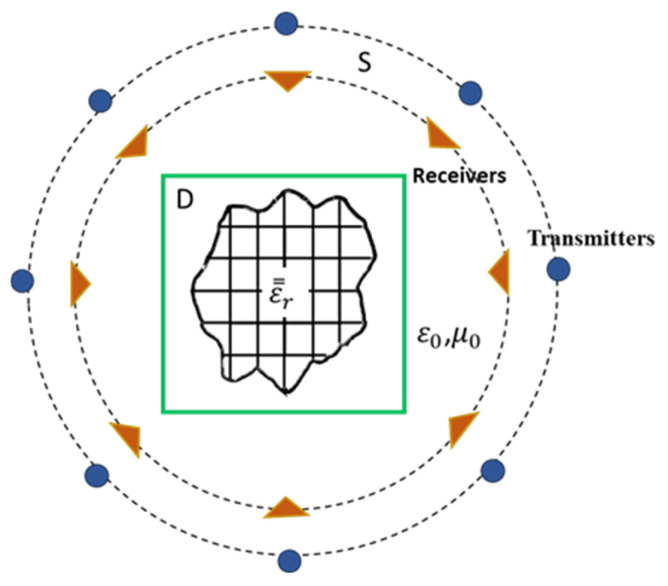
Typical schematic of problem in the plane.

We consider the problem of electromagnetic wave scattering from a two-dimensional uniaxial dielectric object embedded in free space, as illustrated in Figure 1. The scatterer is assumed to be infinitely extended along the *z*-axis, thereby reducing the analysis to a two-dimensional formulation in the transverse x y-plane. The object exhibits anisotropic electromagnetic properties along the cylindrical *z*-axis, and its relative permittivity tensor ${\overset{̿}{\varepsilon }}_{r}$ is defined in the Cartesian coordinate system as follows:

(1) $${{\overset{̿}{\varepsilon }}_{r}=\left[\begin{array}{ccc} \varepsilon_{x}\left(x,y\right) & 0 & 0\\ 0 & \varepsilon_{y}\left(x,y\right) & 0\\ 0 & 0 & \varepsilon_{z}\left(x,y\right) \end{array}\right]}_{xyz}$$

### 2.1. Direct Problem

The object is illuminated by time-harmonic plane waves of the form $e^{j\omega t}$. We consider both TM and TE polarizations. In the TM case, the incident electric field is aligned with the *z*-axis, expressed as

(2) $${\bar{E}}^{i}\left(\overset{⃑}{r}\right)=E_{z}^{i}\left(x,y\right)\hat{z}=e^{-jk_{0}(x\cos\emptyset +y\sin\emptyset )}\hat{z}$$

where $k_{0}$ is the free-space wave number and ∅ is the incident angle. Due to the polarization, the resulting scattered and total fields contain only the z-component. The total field equation is as follows:

(3) $$E_{z}\left(\bar{r}\right)=\int_{s}G(\overset{⃑}{r},\overset{⃑}{r}\prime )(\varepsilon_{z}\left(\overset{⃑}{r}\prime \right)-1)E_{z}\left(\overset{⃑}{r}\prime \right)ds^{\prime }+E_{z}^{i}\left(\overset{⃑}{r}\right),\;\overset{⃑}{r},\overset{⃑}{r}\prime \in S$$

The scattered field observed outside the object is given by

(4) $$E_{z}^{s}\left(\bar{r}\right)=\int_{s}G(\overset{⃑}{r},\overset{⃑}{r}\prime )(\varepsilon_{z}\left(\overset{⃑}{r}\prime \right)-1)E_{z}\left(\overset{⃑}{r}\prime \right)ds^{\prime },\;\overset{⃑}{r}\notin S,\;\overset{⃑}{r}\prime \in S$$

$G\left(\overset{⃑}{r},\overset{⃑}{r}\prime \right)=-\frac{jk_{0}^{2}}{4}H_{0}^{\left(2\right)}\left(k_{0}\left|\overset{⃑}{r}-\overset{⃑}{r}\prime \right|\right)$ is the two-dimensional free space Green’s function.

For TE waves, the incident electric field lies in the transverse x and y-plane:

(5) $$E_{x}^{i}(\overset{⃑}{r})=-\sin\emptyset e^{-jk_{0}(x\cos\emptyset +y\sin\emptyset \;)}$$

(6) $$E_{y}^{i}(\overset{⃑}{r})=\cos\emptyset \;e^{-jk_{0}(x\cos\emptyset +y\sin\emptyset )}$$

Due to the directional dependence of the permittivity, $E_{x}$ and $E_{y}$ become coupled, and their solution requires vectorial treatment. The integral equations for the total fields and scattered fields are as follows:

(7) $$E_{x}\left(\overset{⃑}{r}\right)=\left(\frac{\partial^{2}}{\partial x^{2}}+{k_{0}}^{2}\right)\left[\int_{s}G\left(\overset{⃑}{r},\overset{⃑}{r}\prime \right)\left(\varepsilon_{x}\left(\overset{⃑}{r}\prime \right)-1\right)E_{x}\left(\overset{⃑}{r}\prime \right)ds^{\prime }\right]+\frac{\partial^{2}}{\partial x\partial y}\left[\int_{s}G\left(\overset{⃑}{r},\overset{⃑}{r}\prime \right)\left(\varepsilon_{y}\left(\overset{⃑}{r}\prime \right)-1\right)E_{y}\left(\overset{⃑}{r}\prime \right)ds^{\prime }\right]+{\overset{⃑}{E}}_{x}^{i}\left(\overset{⃑}{r}\right)$$

(8) $$E_{y}\left(\overset{⃑}{r}\right)=\frac{\partial^{2}}{\partial x\partial y}\left[\int_{s}G\left(\overset{⃑}{r},\overset{⃑}{r}\prime \right)\left(\varepsilon_{x}\left(\overset{⃑}{r}\prime \right)-1\right)E_{x}\left(\overset{⃑}{r}\prime \right)ds^{\prime }\right]+\left(\frac{\partial^{2}}{\partial y^{2}}+{k_{0}}^{2}\right)\left[\int_{s}G\left(\overset{⃑}{r},\overset{⃑}{r}\prime \right)\left(\varepsilon_{y}\left(\overset{⃑}{r}\prime \right)-1\right)E_{y}\left(\overset{⃑}{r}\prime \right)ds^{\prime }\right]+{\overset{⃑}{E}}_{y}^{i}\left(\overset{⃑}{r}\right)$$

(9) $$E_{x}^{s}\left(\overset{⃑}{r}\right)=\left(\frac{\partial^{2}}{\partial x^{2}}+{k_{0}}^{2}\right)\left[\int_{s}G\left(\overset{⃑}{r},\overset{⃑}{r}\prime \right)\left(\varepsilon_{x}\left(\overset{⃑}{r}\prime \right)-1\right)E_{x}\left(\overset{⃑}{r}\prime \right)ds^{\prime }\right]+\frac{\partial^{2}}{\partial x\partial y}\left[\int_{s}G\left(\overset{⃑}{r},\overset{⃑}{r}\prime \right)\left(\varepsilon_{y}\left(\overset{⃑}{r}\prime \right)-1\right)E_{y}\left(\overset{⃑}{r}\prime \right)ds^{\prime }\right]$$

(10) $$E_{y}^{s}\left(\overset{⃑}{r}\right)=\frac{\partial^{2}}{\partial x\partial y}\left[\int_{s}G\left(\overset{⃑}{r},\overset{⃑}{r}\prime \right)\left(\varepsilon_{x}\left(\overset{⃑}{r}\prime \right)-1\right)E\hat{x}\left(\overset{⃑}{r}\prime \right)ds^{\prime }\right]+\left(\frac{\partial^{2}}{\partial y^{2}}+{k_{0}}^{2}\right)\left[\int_{s}G\left(\overset{⃑}{r},\overset{⃑}{r}\prime \right)\left(\varepsilon_{y}\left(\overset{⃑}{r}\prime \right)-1\right)E\hat{y}\left(\overset{⃑}{r}\prime \right)ds^{\prime }\right]$$

To facilitate numerical analysis, the scatterer is discretized into N small uniform cells. Using the Method of Moments (MoM), the integral equations are transformed into linear systems by expanding the fields using pulse basis functions and testing with delta functions. The equations are transformed into matrix form as follows:

(11) $$-\left(E_{z}^{i}\right)=\left(\left[G_{1}\right]\left[\tau_{z}\right]-\left[I\right]\right)\left(E_{z}\right)$$

(12) $$\left(E_{z}^{s}\right)=\left[G_{2}\right]\left[\tau_{z}\right]\left(E_{z}\right)$$

(13) $$\left(\begin{array}{c} {-E}_{x}^{i}\\ {-E}_{y}^{i} \end{array}\right)=\left\{\left[\begin{array}{cc} \left[G_{3}\right] & \left[G_{4}\right]\\ \left[G_{4}\right] & \left[G_{5}\right] \end{array}\right]\left[\begin{array}{cc} \left[\tau_{x}\right] & 0\\ 0 & \left[\tau_{y}\right] \end{array}\right]-\left[\begin{array}{cc} \left[I\right] & 0\\ 0 & \left[I\right] \end{array}\right]\right\}\left(\begin{array}{c} E_{x}\\ E_{y} \end{array}\right)$$

(14) $$\left(\begin{array}{c} E_{x}^{s}\\ E_{y}^{s} \end{array}\right)=\left\{\left[\begin{array}{cc} \left[G_{6}\right] & \left[G_{7}\right]\\ \left[G_{7}\right] & \left[G_{8}\right] \end{array}\right]\left[\begin{array}{cc} \left[\tau_{x}\right] & 0\\ 0 & \left[\tau_{y}\right] \end{array}\right]\right\}\left(\begin{array}{c} E_{x}\\ E_{y} \end{array}\right)$$

The Green’s function matrix can be expressed as

(15) $${\left(G_{1}\right)}_{mn}=\left\{\begin{array}{c} -\frac{j\pi k_{0}a_{n}}{2}J_{1}\left(k_{0}a_{n}\right){H_{0}}^{\left(2\right)}\left(k_{0}\rho_{mn}\right)\;,\;m\neq n\\ -\frac{j}{2}\left[{\pi k}_{0}a_{n}{H_{1}}^{\left(2\right)}\;\left(k_{0}a_{n}\right)-2j\right]\;,\;m=n \end{array}\right.$$

(16) $${\left(G_{2}\right)}_{mn}=-\frac{j\pi k_{0}a_{n}}{2}J_{1}\left(k_{0}a_{n}\right){H_{0}}^{\left(2\right)}\left(k_{0}\rho_{mn}\right)$$

(17) $$\left(G_{3}\right)=\left\{\begin{array}{c} -\frac{j\pi a_{n}J_{1}\left(k_{0}a_{n}\right)}{2{\rho^{3}}_{mn}}\times [k\rho_{mn}{\left(y_{m}-y_{n}\right)}^{2}{H_{0}}^{\left(2\right)}(k_{0}\rho_{mn})+\\ \left({\left(x_{m}-x_{n}\right)}^{2}-{\left(y_{m}-y_{n}\right)}^{2}){H_{1}}^{\left(2\right)}(k_{0}\rho_{mn}\right)]\;,m\neq n\\ \;-\frac{j}{4}\left[\pi k_{0}a_{n}{H_{1}}^{\left(2\right)}\left(k_{0}a_{n}\right)-4j\right]\;,\;m=n \end{array}\right\}$$

(18) $$\left(G_{4}\right)=\left\{\begin{array}{c} \;-\frac{j\pi a_{n}J_{1}\left(k_{0}a_{n}\right)}{2{\rho^{3}}_{mn}}(x_{m}-x_{n})(y_{m}-y_{n})\times \\ \begin{array}{cc} \left[2{H_{1}}^{\left(2\right)}\left(k_{0}\rho_{mn}\right)-k_{0}\rho_{mn}{H_{0}}^{\left(2\right)}\left(k_{0}\rho_{mn}\right)\right] & ,m\neq n \end{array}\\ 0\;,\;m=n \end{array}\right\}$$

(19) $$\left(G_{5}\right)=\left\{\begin{array}{c} -\frac{j\pi a_{n}J_{1}\left(k_{0}a_{n}\right)}{2{\rho^{3}}_{mn}}\times [k_{0}\rho_{mn}{\left(x_{m}-x_{n}\right)}^{2}{H_{0}}^{\left(2\right)}(k_{0}\rho_{mn})+)\\ \;\left({\left(y_{m}-y_{n}\right)}^{2}-{\left(x_{m}-x_{n}\right)}^{2}\right)]\;,m\neq n\\ \;-\frac{j}{4}[\pi k_{0}a_{n}{H_{1}}^{\left(2\right)}\left(k_{0}a_{n}\right)-4j]\;,\;m=n \end{array}\right\}$$

(20) $$\begin{array}{c} \left(G_{6}\right)=-\frac{j\pi a_{n}J_{1}\left(k_{0}a_{n}\right)}{2{\rho^{3}}_{mn}}\\ \;\times [k_{0}\rho_{mn}{\left(y_{m}-y_{n}\right)}^{2}{H_{0}}^{\left(2\right)}\left(k_{0}\rho_{mn}\right)\\ \;+({\left(x_{m}-x_{n}\right)}^{2}-{\left(y_{m}-y_{n}\right)}^{2}){H_{1}}^{\left(2\right)}(k_{0}\rho_{mn})] \end{array}$$

(21) $${\left(G_{7}\right)}_{mn}=-\frac{j\pi a_{n}J_{1}\left(k_{0}a_{n}\right)}{2{\rho^{3}}_{mn}}\left(x_{m}-x_{n}\right)\left(y_{m}-y_{n}\right)\left[2{H_{1}}^{\left(2\right)}\left(k_{0}\rho_{mn}\right)-k_{0}\rho_{mn}{H_{0}}^{\left(2\right)}\left(k_{0}\rho_{mn}\right)\right]$$

(22) $${\left(G_{8}\right)}_{mn}=-\frac{j\pi a_{n}J_{1}\left(k_{0}a_{n}\right)}{2{\rho^{3}}_{mn}}[k_{0}\rho_{mn}{\left(x_{m}-x_{n}\right)}^{2}{H_{0}}^{\left(2\right)}\left(k_{0}\rho_{mn}\right)+({\left(y_{m}-y_{n}\right)}^{2}-{\left(x_{m}-x_{n}\right)}^{2}){H_{1}}^{\left(2\right)}(k_{0}\rho_{mn})]$$

Here, $\rho_{mn}=\sqrt{{\left(x_{m}-\;x_{n}\right)}^{2}+{\left(y_{m}-y_{n}\right)}^{2}}$ denotes the Euclidean distance between the m-th observation point and the n-th source point. $H_{0}^{\left(2\right)}$ and ${H_{1}}^{\left(2\right)}$ represent the zero-order and first-order Hankel functions of the second kind, respectively, while $J_{1}$ denotes the first-order Bessel function.

### 2.2. Inverse Problem

To obtain a physically meaningful initial estimate of the dielectric distribution, we adopt the BPS, which is particularly effective in reconstructing weakly scattering objects. The key idea is to estimate the induced current from the measured scattered field and then retrieve the contrast function from this induced current. For the TM case, the induced current is assumed to be proportional to the scattered field via the conjugate transpose of the Green’s function matrix:

(23) $$(I_{z}^{b})=Y_{m}\cdot {\left[G_{2}\right]}^{H}\left(E_{z}^{s}\right)$$

where $Y_{m}$ is a scalar coefficient to be optimized, ${\left[G_{2}\right]}^{H}$ denotes the Hermitian transpose of the Green’s function matrix, and $E_{z}^{s}$ is the measured scattered field. The corresponding loss function can be defined as the discrepancy between the measured field and the field synthesized from the induced current:

(24) $$L_{m}^{b}(Y)={\left\|\left(E_{z}^{s}\right)-\left[G_{2}\right]\cdot Y_{m}\cdot {\left[G_{2}\right]}^{H}\left(E_{z}^{s}\right)\right\|}^{2}$$

Minimizing this function with respect to $Y_{m}$ yields the following analytical solution:

(25) $$Y_{m}=\frac{{\left(E_{z}^{s}\right)}^{T}\cdot {\left(\left[G_{2}\right]\left({\left[G_{2}\right]}^{H}\cdot \left(E_{z}^{s}\right)\right)\right)}^{*}}{{\left\|\left[G_{2}\right]\left({\left[G_{2}\right]}^{H}\cdot \left(E_{z}^{s}\right)\right)\right\|}^{2}}$$

With $Y_{m}$ determined, the total field in the object region can be estimated by

(26) $$\left(E_{z}^{b})=(E_{z}^{i})+[G_{1}](I_{z}^{b}\right)$$

Assuming the induced current is the product of the contrast and the total field,

(27) $$\left(I_{z,p}^{b}\right)=diag\left(\left[\tau_{z}^{b}\right]\right)\left(E_{z}^{b}\right)$$

The contrast $\left[\tau_{z}^{b}\right]$ can then be estimated in a least-squares sense over multiple incident fields:

(28) $$\left[\tau_{z}^{b}\right]=\frac{\sum_{p=1}^{Ni}I_{z,p}^{b}\left(n\right)\cdot {\left[E_{z}^{b}\left(n\right)\right]}^{*}}{\sum_{p=1}^{Ni}{\left\|E_{z}^{b}\left(n\right)\right\|}^{2}}$$

For the TE case, which involves two components, $E_{x}$ and $E_{y}$, the back-propagated induced current $I_{x}^{b}$ and $I_{y}^{b}$ is computed similarly:

(29) $$\left(\begin{array}{c} I_{x}^{b}\\ I_{y}^{b} \end{array}\right)=Y_{e}\cdot {\left[\begin{array}{cc} \left[G_{6}\right] & \left[G_{7}\right]\\ \left[G_{7}\right] & \left[G_{8}\right] \end{array}\right]}^{H}\left(\begin{array}{c} E_{x}^{s}\\ E_{y}^{s} \end{array}\right)$$

and the associated loss function becomes:

(30) $$L_{e}^{b}(Y)={\left\|\left(\begin{array}{c} E_{x}^{s}\\ E_{y}^{s} \end{array}\right)-\left[\begin{array}{cc} \left[G_{6}\right] & \left[G_{7}\right]\\ \left[G_{7}\right] & \left[G_{8}\right] \end{array}\right]\cdot Y_{e}\cdot {\left[\begin{array}{cc} \left[G_{6}\right] & \left[G_{7}\right]\\ \left[G_{7}\right] & \left[G_{8}\right] \end{array}\right]}^{H}\left(\begin{array}{c} E_{x}^{s}\\ E_{y}^{s} \end{array}\right)\right\|}^{2}$$

The following analytical solution of ${ϒ}_{e}$ can be obtained:

(31) $$Y_{e}=\frac{{\left(\begin{array}{c} E_{x}^{s}\\ E_{y}^{s} \end{array}\right)}^{T}\cdot {\left(\left[\begin{array}{cc} \left[G_{6}\right] & \left[G_{7}\right]\\ \left[G_{7}\right] & \left[G_{8}\right] \end{array}\right]\left({\left[\begin{array}{cc} \left[G_{6}\right] & \left[G_{7}\right]\\ \left[G_{7}\right] & \left[G_{8}\right] \end{array}\right]}^{H}\cdot \left(\begin{array}{c} E_{x}^{s}\\ E_{y}^{s} \end{array}\right)\right)\right)}^{*}}{{\left\|\left[\begin{array}{cc} \left[G_{6}\right] & \left[G_{7}\right]\\ \left[G_{7}\right] & \left[G_{8}\right] \end{array}\right]\left({\left[\begin{array}{cc} \left[G_{6}\right] & \left[G_{7}\right]\\ \left[G_{7}\right] & \left[G_{8}\right] \end{array}\right]}^{H}\cdot \left(\begin{array}{c} E_{x}^{s}\\ E_{y}^{s} \end{array}\right)\right)\right\|}^{2}}$$

The total back-propagated field is then reconstructed as

(32) $$\left(\begin{array}{c} E_{x}^{b}\\ E_{y}^{b} \end{array}\right)=\left(\begin{array}{c} E_{x}^{i}\\ E_{y}^{i} \end{array}\right)+\left[\begin{array}{cc} \left[G_{3}\right] & \left[G_{4}\right]\\ \left[G_{4}\right] & \left[G_{5}\right] \end{array}\right]\left(\begin{array}{c} I_{x}^{b}\\ I_{y}^{b} \end{array}\right)$$

Subsequently, the dielectric contrast is recovered via

(33) $$\left(\begin{array}{c} I_{x,p}^{b}\\ I_{y,p}^{b} \end{array}\right)=diag\left(\left[\begin{array}{cc} \left[\tau_{x}^{b}\right] & 0\\ 0 & \left[\tau_{y}^{b}\right] \end{array}\right]\right)\left(\begin{array}{c} E_{x}^{b}\\ E_{y}^{b} \end{array}\right)$$

By aggregating the results from all illuminations, the least-squares solution for each pixel is obtained as

(34) $$\left[\begin{array}{cc} \left[\tau_{x}^{b}\right] & 0\\ 0 & \left[\tau_{y}^{b}\right] \end{array}\right]=\frac{\sum_{p=1}^{Ni}\begin{array}{c} I_{x,p}^{b}\left(n\right)\\ I_{y,p}^{b}\left(n\right) \end{array}\cdot {\left[\left(\begin{array}{c} E_{x}^{b}\\ E_{y}^{b} \end{array}\right)\left(n\right)\right]}^{*}}{\sum_{p=1}^{Ni}{\left\|\begin{array}{c} E_{x}^{b}\left(n\right)\\ E_{y}^{b}\left(n\right) \end{array}\right\|}^{2}}$$

The BPS framework serves as a physically meaningful and computationally efficient method to generate coarse estimates of the permittivity distribution. These estimates are subsequently used as input to the neural network, enabling the learning model to focus on refining spatial details and improving reconstruction accuracy based on physically informed priors.

## 3. PGAN

**Figure 2:**
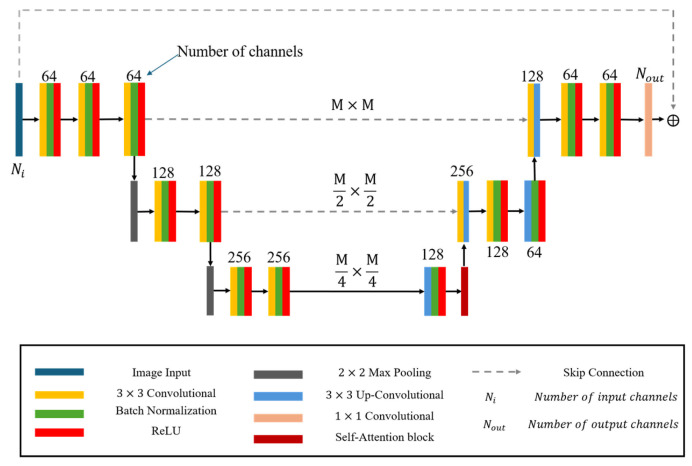
Generator of PGAN.

**Figure 3:**
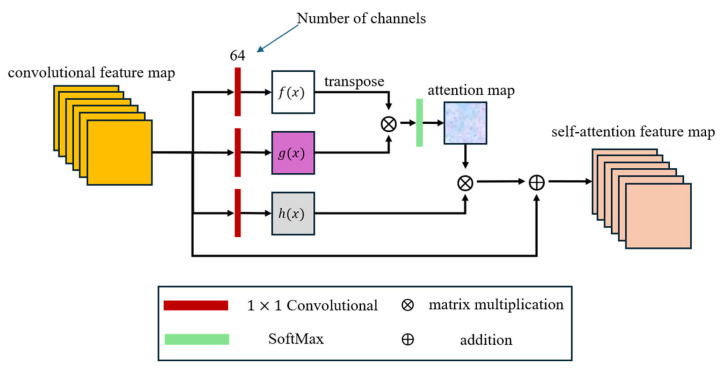
Self-Attention block.

**Figure 4:**
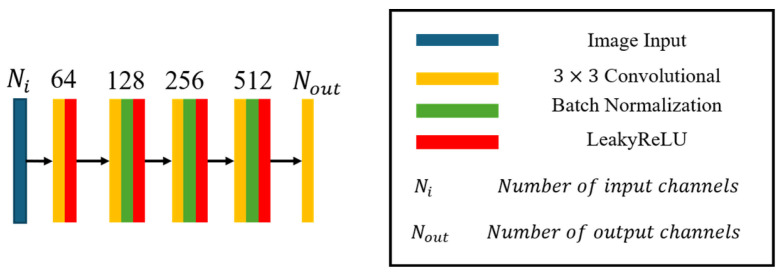
Discriminator of PGAN.

To enhance both the fidelity and resolution of reconstructed images in electromagnetic inverse scattering, we propose a PGAN that integrates spatial feature recovery with perceptual quality optimization. The PGAN generator, illustrated in Figure 2, adopts an encoder–decoder architecture, in which each layer comprises a 3×3 convolution followed by batch normalization and a ReLU activation. The encoder compresses the spatial dimensions while capturing high-level semantic features, whereas the decoder progressively restores fine-grained spatial details through 3×3 transposed convolutions and skip connections, ultimately producing a refined dielectric contrast distribution. To further enhance structural consistency and global contextual awareness, self-attention modules, as shown in Figure 3, are incorporated at the junction between the encoder and decoder, as well as at selected stages along the decoding path. The self-attention module is designed to capture long-range spatial dependencies, thereby enabling the network to more effectively resolve complex scattering patterns and directional features inherent to anisotropic media. The discriminator, illustrated in Figure 4, is constructed to differentiate between the reconstructed dielectric contrast maps generated by the PGAN generator and the corresponding ground truth distributions. It follows a fully convolutional architecture composed of five sequential convolutional blocks, each consisting of a convolutional layer, batch normalization, and LeakyReLU activation. This design promotes stable training and mitigates issues related to vanishing gradients.

To ensure that the reconstructed dielectric contrast maps are both numerically accurate and perceptually coherent, we formulate a composite loss function for the PGAN generator that integrates pixel-level fidelity, perceptual similarity, and semantic alignment. The total generator loss is defined as

(35) $$L_{G}=L_{1}+\beta L_{A}+\gamma L_{PA}+\eta (1-SSIM)$$

(36) $$L_{1}={\left\|G_{\theta }-\varepsilon \right\|}_{1}$$

(37) $$L_{A}={\left\|D_{\phi }\left(\varepsilon \prime ,G_{\theta }\left(\varepsilon \prime \right)\right)-D_{\phi }\left(\varepsilon \prime ,\varepsilon \right)\right\|}_{1}$$

(38) $$L_{PA}=\sum_{j=1}^{M_{d}}{\left\|d_{j,\;\emptyset }\left(\varepsilon \prime ,G_{\theta }\left(\varepsilon \prime \right)\right)-d_{j,\;\emptyset }\left(\varepsilon \prime ,\varepsilon \right)\right\|}_{1}$$

Here, ε′ denotes the initial guess by BPS, ε denote ground truth and $L_{1}$ denotes the pixel-wise reconstruction error measured by the $L_{1}$-norm, which ensures numerical fidelity between the generated and ground-truth dielectric distributions. The coefficients β, γ and η are weighting parameters that control the relative influence of the semantic consistency term $L_{A}$, the perceptual adversarial loss $L_{PA}$ and the SSIM-related structural loss, respectively. Specifically, $L_{PA}$ is designed to guide the generator toward producing perceptually realistic images by comparing intermediate feature representations between synthesized and ground-truth samples, as extracted by the discriminator. The inclusion of these complementary loss components allows the network to balance pixel-level accuracy with global perceptual and semantic coherence during training.

To effectively differentiate the predicted dielectric profiles from the true distributions, the discriminator is optimized using a least-squares loss function. The discriminator loss function $L_{D}$ is given by

(39) $$L_{D}=\frac{1}{2}\left({\left\|D_{\phi }(\varepsilon \prime ,\varepsilon )-1\right\|}_{2}^{2}+{\left\|D_{\phi }\left(\varepsilon \prime ,G_{\theta }\left(\varepsilon \prime \right)\right)\right\|}_{2}^{2}\right)$$

where $D_{\phi }$ denotes the output of the discriminator parameterized by ϕ. The first term encourages the discriminator to assign a high confidence (close to 1) to real samples, while the second term penalizes the discriminator if it fails to identify the generated samples as fake. This design facilitates more stable and efficient adversarial training, particularly for complex imaging tasks such as electromagnetic inverse scattering involving anisotropic objects.

## 4. Numerical Results

In the numerical simulations, the anisotropic dielectric targets are considered in a free-space imaging domain, with the transmitting and receiving antennas uniformly arranged around the region of interest. The targets are illuminated from multiple incident directions using both TM and TE polarizations, and the corresponding scattered-field responses are collected at the receiver locations. Rather than using the scattered-field data directly as the input to the neural network, BPS is first employed to generate a physics-based preliminary reconstruction of the relative permittivity distribution, which is subsequently used as the input to the PGAN with SA for further refinement. The computational domain is discretized with a spatial interval of $\frac{0.2\lambda_{0}}{\sqrt{\varepsilon_{r}}}$, where $\lambda_{0}$ denotes the free-space wavelength and $\varepsilon_{r}$ denotes the relative permittivity of the target. In the simulations, the relative permittivity ranges from 1 to 2.5, the operating frequency is fixed at 3 GHz, and 32 transmitters and 32 receivers are uniformly distributed around the imaging region. To evaluate the robustness of the reconstruction results against noise interference, Gaussian noise with amplitudes of 5% and 20% is added to the simulated scattered-field data.

For implementation, all BPS-reconstructed dielectric profiles are represented as 32×32 images and used as the input to the PGAN with SA, while the corresponding ground truth permittivity distributions with the same image size are used as the output labels. For numerical results, each dataset contains 500 samples, generated from 10 dielectric profiles randomly placed at 50 different locations. For the experimental FoamDielExt case, the training dataset is generated using 15,120 combinations of dielectric constant ranges, and the trained model is then evaluated using the measured data from the Fresnel Institute database. Before being fed into the network, the BPS images and ground truth labels are normalized to [0, 1] according to their corresponding permittivity ranges and then mapped back to the physical permittivity values for quantitative evaluation. For the generator loss function defined in Equation (35), the weighting coefficients are set to β=0.01, γ=1, and η=20 for all experiments. The dielectric distributions estimated by BPS are divided into training and testing sets, with 80% of the data used for training, 10% for validation and the remaining 10% for testing. The PGAN model is optimized using the adaptive moment estimation (ADAM) optimizer. The exponential decay rates for the moment estimates are set to $\beta_{1}=0.9$ and $\beta_{2}=0.99$. The learning rate is initialized at $2\times 10^{-4}$ for the first 30 epochs and increased to $4\times 10^{-4}$ thereafter. A batch size of 32 is used, and training is conducted for a maximum of 40 epochs. All experiments are implemented in MATLAB R2025b and performed on a workstation equipped with an Intel 14th-generation Core i7 CPU, an NVIDIA RTX 40-series GPU, and 128 GB RAM.

The reconstruction performance is quantitatively evaluated using RMSE and SSIM. The RMSE is adopted to measure the normalized numerical discrepancy between the reconstructed and ground-truth relative permittivity tensors and is defined as

(40) $$RMSE=\frac{1}{M_{t}}{\sum_{i=1}^{M_{t}}\left\|{\overset{̿}{\varepsilon }}_{r}-{\overset{̿}{\varepsilon }}_{r}^{r}\right\|}_{F}/{\left\|{\overset{̿}{\varepsilon }}_{r}\right\|}_{F}$$

where ${\overset{̿}{\varepsilon }}_{r}$ and ${\overset{̿}{\varepsilon }}_{r}^{r}$ represent the reconstructed and true permittivity distributions, respectively; $M_{t}$ denotes the number of test samples; and F denotes the Frobenius norm.

The SSIM metric is further used to assess the structural consistency between the reconstructed and ground-truth permittivity profiles and is defined as

(41) $$SSIM=\frac{\left(2\mu_{\tilde{y}}\mu_{y}+C_{1})(2\sigma_{\tilde{y}y}+C_{2}\right)}{\left(\mu_{\tilde{y}}^{2}+\mu_{y}^{2}+C_{1})(\sigma_{\tilde{y}}^{2}+\sigma_{y}^{2}+C_{2}\right)}$$

Here, $\tilde{y}$ and y denote the reconstructed and ground-truth relative permittivity profiles, respectively. $\mu_{y}$ is the mean of y, $\sigma_{y}^{2}$ is the variance of $\tilde{y}$, and $\sigma_{\tilde{y}y}$ represents the covariance between $\tilde{y}$ and y. To prevent division by zero, two small constants are introduced: $C_{1}={\left(K_{1}D\right)}^{2}$ and $C_{2}={\left(K_{2}D\right)}^{2}$, where $K_{1}=0.01$ and $K_{2}=0.03$ are empirically chosen hyperparameters.

### 4.1. Reconstruction of Homogeneous Dielectric Object with a Relative Permittivity Ranging from 1 to 1.5

In this scenario, the relative permittivity distribution is configured within the range of 1.0 to 1.5. A total of 32 transmitters and 32 receivers are uniformly arranged around the imaging domain. To more realistically simulate practical measurement conditions, 20% Gaussian noise is added to the environment to evaluate the robustness of the reconstruction models. The scatterers are defined by 10 distinct permittivity profiles, each randomly positioned at 50 different locations within the observation region. Consequently, the dataset comprises 500 images (10 profiles × 50 placements), which are divided into 80% for training, 10% for validation, and 10% for testing. To reduce the complexity of the network training process, the measured scattered fields are first processed through the BPS to obtain a coarse estimation of the dielectric distribution. These initial estimates are then fed into the proposed PGAN model to reconstruct a high-fidelity dielectric contrast map.

**Figure 5:**
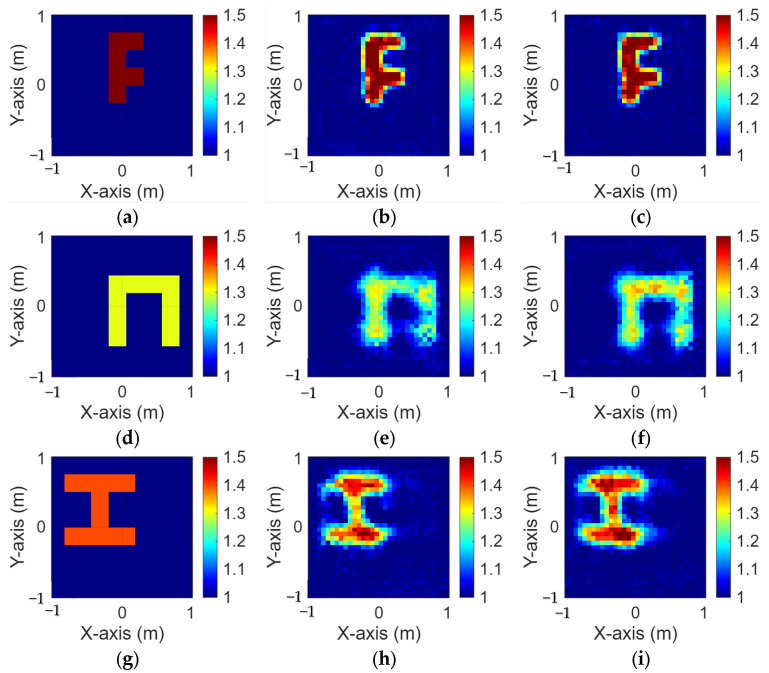
Reconstruction results of a homogeneous anisotropic object with relative permittivity ranging from 1 to 1.5 under 20% Gaussian noise. (**a**) Ground Truth for $\varepsilon_{z}$. (**b**) PGAN reconstruction for $\varepsilon_{z}$. (**c**) PGAN reconstruction with SA for $\varepsilon_{z}$. (**d**) Ground Truth for $\varepsilon_{x}$. (**e**) PGAN reconstruction for $\varepsilon_{x}$. (**f**) PGAN reconstruction with SA for $\varepsilon_{x}$. (**g**) Ground Truth for $\varepsilon_{y}$. (**h**) PGAN reconstruction for $\varepsilon_{y}.$ (**i**) PGAN reconstruction with SA for $\varepsilon_{y}$.

Figure 5 compares the reconstruction results of the anisotropic permittivity components $\varepsilon_{z}$, $\varepsilon_{x}$, and $\varepsilon_{y}$ obtained by PGAN and PGAN with SA. Although both methods can recover the main spatial distributions of the dielectric profiles, PGAN with SA consistently produces reconstructions with superior visual quality for all three components. Compared with the original PGAN, the incorporation of self-attention leads to improved structural fidelity, sharper boundary delineation, and reduced reconstruction artifacts, indicating a stronger capability for reconstructing anisotropic permittivity distributions. This trend is also consistently reflected in the quantitative results listed in Table 1. The RMSE decreases from 4.45% to 4.28% for $\varepsilon_{z}$, from 5.06% to 4.72% for $\varepsilon_{x}$, and from 6.54% to 6.1266% for $\varepsilon_{y}$. At the same time, the SSIM increases from 91.19% to 91.63%, from 78.47% to 80.84%, and from 76.45% to 79.59%, respectively. These quantitative and visual results consistently verify that self-attention enhances both reconstruction accuracy and structural consistency, thereby improving the robustness of anisotropic dielectric image reconstruction under strong noise.

### 4.2. Reconstruction of Inhomogeneous Dielectric Object with a Relative Permittivity Ranging from 1.5 to 2

For this case, the targets are modeled as inhomogeneous dielectric distributions with relative permittivity values ranging from 1.5 to 2.0. The imaging system employs 32 uniformly distributed transmitters and 32 receivers around the investigation domain. To evaluate the robustness of the proposed method under realistic conditions, 5% Gaussian noise is added to the measurement data. The dataset contains 10 distinct inhomogeneous permittivity profiles, each placed at 50 random positions, yielding a total of 500 samples. The samples are partitioned into training, validation, and testing sets in an 80:10:10 ratio. To simplify the learning task, the scattered-field measurements are first transformed by the BPS into coarse dielectric estimates, which are then used as the input to the proposed PGAN for high-fidelity reconstruction.

**Figure 6:**
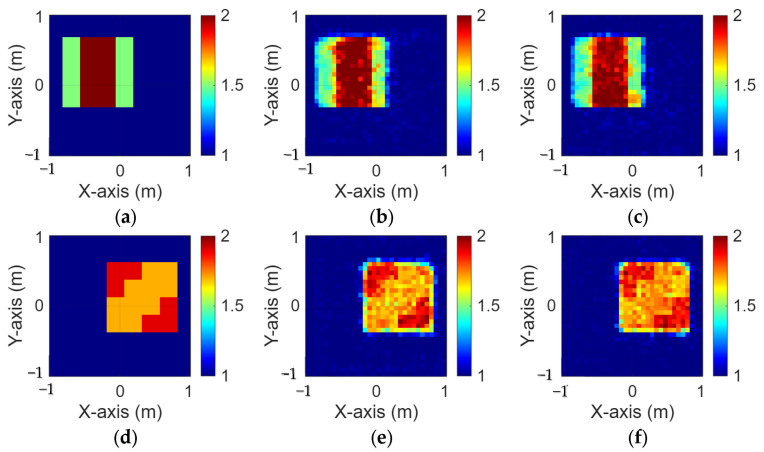

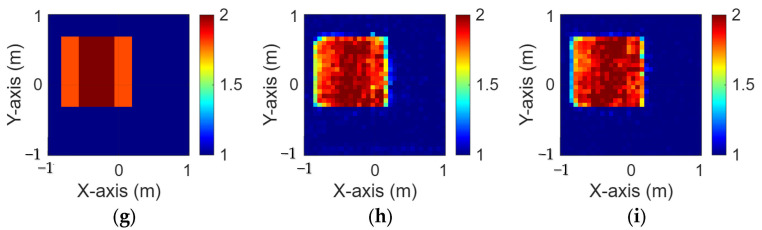
Reconstruction results of an inhomogeneous anisotropic object with relative permittivity ranging from 1.5 to 2 under 5% Gaussian noise. (**a**) Ground Truth for $\varepsilon_{z}$. (**b**) PGAN reconstruction for $\varepsilon_{z}$. (**c**) PGAN reconstruction with SA for $\varepsilon_{z}$. (**d**) Ground Truth for $\varepsilon_{x}$. (**e**) PGAN reconstruction for $\varepsilon_{x}$. (**f**) PGAN reconstruction with SA for $\varepsilon_{x}$. (**g**) Ground Truth for $\varepsilon_{y}$. (**h**) PGAN reconstruction for $\varepsilon_{y}$. (**i**) PGAN reconstruction with SA for $\varepsilon_{y}$.

Figure 6 compares the reconstruction results of the anisotropic permittivity components $\varepsilon_{z}$, $\varepsilon_{x}$, and $\varepsilon_{y}$ for an inhomogeneous anisotropic object using PGAN and PGAN with SA. The visual results show that, while both methods can recover the overall target regions, PGAN with SA yields reconstructions that are closer to the ground truth not only in the object contour but also in the internal inhomogeneous permittivity distribution. Compared with the original PGAN, the proposed PGAN with SA better preserves regional dielectric variations, maintains clearer transitions between different permittivity levels, and suppresses undesired reconstruction artifacts. This demonstrates that the integration of self-attention improves the ability of PGAN to reconstruct inhomogeneous anisotropic permittivity distributions with improved structural fidelity and internal contrast consistency. The quantitative results listed in Table 2 further confirm the advantage of PGAN with SA over the original PGAN for the reconstruction of inhomogeneous anisotropic dielectric objects. The RMSE decreases from 8.46% to 7.93% for $\varepsilon_{z}$, from 6.63% to 5.08% for $\varepsilon_{x}$, and from 7.25% to 5.68% for $\varepsilon_{y}$. At the same time, the SSIM increases from 88.7% to 89.21%, from 92.13% to 95.6%, and from 92.08% to 95.59%, respectively. These quantitative improvements verify that self-attention enhances not only reconstruction accuracy but also the preservation of structural characteristics in the recovered dielectric profiles. Together with the visual results in Figure 6, these findings confirm that PGAN with SA provides a more reliable framework for reconstructing inhomogeneous anisotropic permittivity distributions.

### 4.3. Reconstruct the Modified National Institute of Standards and Technology (MNIST) Database with Relative Permittivity Ranging from 2 to 2.5

For this case, the reconstruction targets are generated from the Modified National Institute of Standards and Technology (MNIST) database, with relative permittivity values ranging from 2.0 to 2.5. The imaging system employs 32 transmitters and 32 receivers uniformly distributed around the investigation domain. To evaluate the robustness of the proposed method under practical conditions, 5% Gaussian noise is added to the measurement data. The dataset consists of 10 distinct MNIST-based dielectric patterns, each randomly placed at 50 different locations, resulting in a total of 500 samples. The samples are divided into training, validation, and testing sets with a ratio of 80:10:10. To simplify the reconstruction task, the scattered-field measurements are first processed using BPS to obtain coarse dielectric estimates, which are subsequently fed into the proposed PGAN for high-fidelity reconstruction.

**Figure 7:**
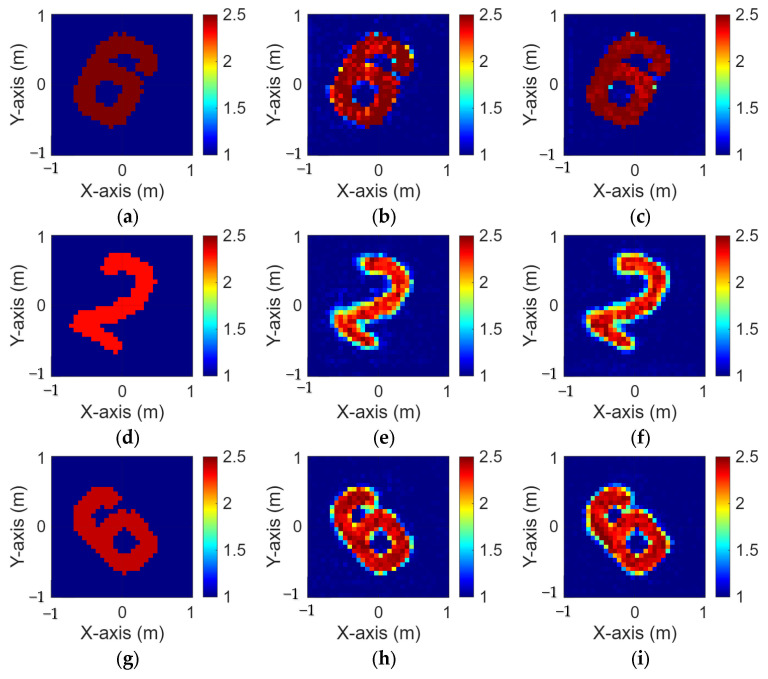
Reconstruction results of MNIST with relative permittivity ranging from 2 to 2.5 under 5% Gaussian noise. (**a**) Ground Truth for $\varepsilon_{z}$. (**b**) PGAN reconstruction for $\varepsilon_{z}$. (**c**) PGAN reconstruction with SA for $\varepsilon_{z}$. (**d**) Ground Truth for $\varepsilon_{x}$. (**e**) PGAN reconstruction for $\varepsilon_{x}$. (**f**) PGAN reconstruction with SA for $\varepsilon_{x}$. (**g**) Ground Truth for $\varepsilon_{y.}$ (**h**) PGAN reconstruction for $\varepsilon_{y}$. (**i**) PGAN reconstruction with SA for $\varepsilon_{y}$.

Figure 7 compares the reconstruction results of the anisotropic permittivity components $\varepsilon_{z}$, $\varepsilon_{x}$, and $\varepsilon_{y}$ for MNIST-based dielectric targets obtained by PGAN and PGAN with SA. Although both methods can recover the overall target regions, PGAN with SA yields reconstructions that are consistently closer to the ground truth in terms of contour accuracy, structural integrity, and internal dielectric consistency. Compared with the original PGAN, the self-attention mechanism enables better preservation of curved boundaries and local geometric details, while also suppressing undesired reconstruction artifacts in the background region. This indicates that PGAN with SA is more effective in reconstructing anisotropic permittivity distributions of geometrically complex targets, thereby providing improved robustness and visual fidelity. The quantitative results listed in Table 3 further support the advantage of PGAN with SA over the original PGAN for MNIST-based anisotropic dielectric reconstruction. The RMSE decreases from 10.77% to 10.27% for $\varepsilon_{z}$, from 11.83% to 10.89% for $\varepsilon_{x}$, and from 12.5% to 11.3% for $\varepsilon_{y}$. At the same time, SSIM increases from 85.59% to 86.96%, from 83.9% to 88%, and from 84.02% to 87.83%, respectively. These results indicate that self-attention enhances not only the reconstruction accuracy but also the preservation of structural characteristics in the recovered dielectric profiles. Together with the visual results in Figure 7, these findings confirm that PGAN with SA provides a more reliable framework for reconstructing anisotropic dielectric targets with complex spatial geometries.

**Figure 8:**
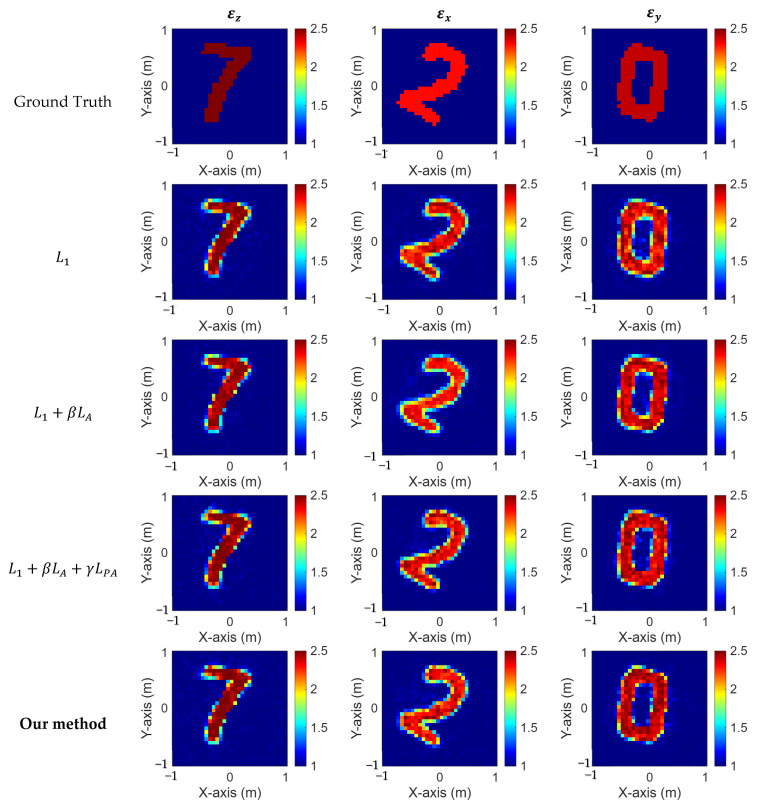
Comparing the performance of different loss functions.

To further evaluate the contribution of the loss-function design, different loss-function settings were compared by using the PGAN with SA architecture. The reconstruction results are shown in Figure 8, and the corresponding quantitative performance is summarized in Table 4. It can be observed that adding the SSIM term provides the best performance among the compared loss function in terms of reconstruction accuracy and structural similarity. Compared with the models trained without the SSIM-related term, our proposed loss function $L_{1}+\beta L_{A}+\gamma L_{PA}+(1-SSIM)$ achieves lower RMSE and higher SSIM. This indicates that the SSIM-related structural loss is effective in preserving local structural information, boundary consistency, and geometric details in the reconstructed anisotropic dielectric profiles. Therefore, the hybrid loss function incorporating the SSIM term was adopted for all experiments in this paper.

### 4.4. Reconstructing the Experimental Database

**Figure 9:**
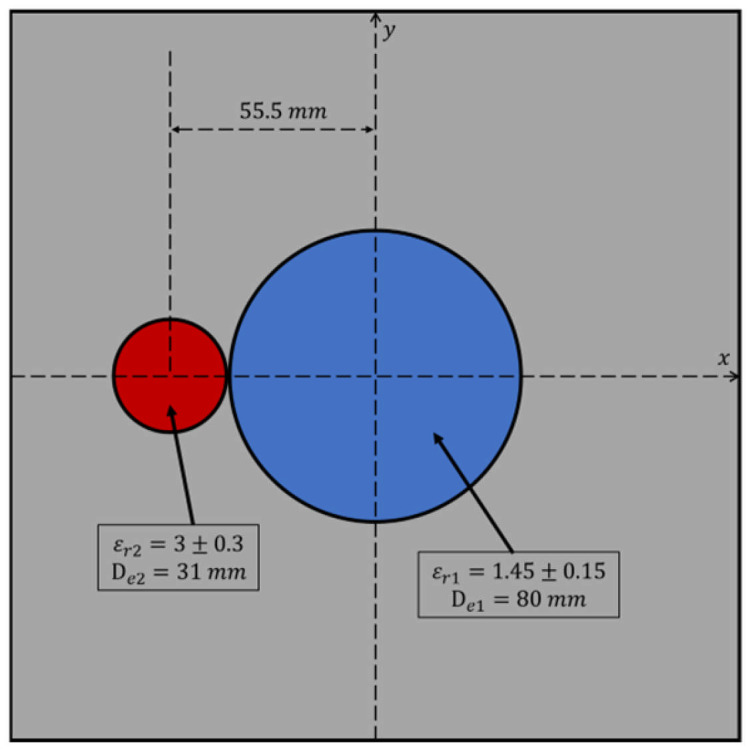
The schematic diagram of the FoamDielExt object is presented here, where “±” indicates the experimental uncertainty range and $D_{e}$ denotes the diameter.

The training dataset was constructed in a similar manner to the previous case, except that the relative permittivity range was extended to 1.3–3.3. In this setting, eight transmitters are employed, resulting in a total of 15,120 images (1890 × 8) for network training. Because the noise characteristics of the experimental measurements are not available in advance, the networks are trained using noise-free synthetic data and then evaluated with measured experimental data. In this work, the experimental dataset released by the Fresnel Institute [20] is adopted to assess the reconstruction performance of U-Net, PGAN, and PGAN with SA under practical measurement conditions. For experimental measurements, the dataset includes 8 transmitters and 241 receivers. The distance between the source and the scatterer is 1.67 m. Since horn antennas are used to collect the scattered fields, the receivers are not located on both sides of the transmitting antenna. The selected target is FoamDielExt, considered for both the TM and TE polarizations. This object is composed of a large cylinder made of SAITEC SBF 300 material and a small cylinder made of Berlon material. Their diameters are 80 mm and 31 mm, respectively, with corresponding relative permittivity values of $\varepsilon_{r}=1.45\pm 0.15$ and $\varepsilon_{r}=3.0\pm 0.3$. A schematic of the FoamDielExt object is presented in Figure 9. In the simulation setting, the scatterer is located within a 320 mm × 320 mm domain, and the operating frequency of the incident wave is fixed at 3 GHz for both the TM and TE cases. To ensure consistency between simulation and experiment, the simulated scattered fields observed on the side opposite to the incident direction are used to normalize the measured data. The normalized experimental data are then provided as the input to U-Net, PGAN and PGAN with SA for dielectric profile reconstruction, allowing a direct evaluation of the two methods on experimental measurements.

**Figure 10:**
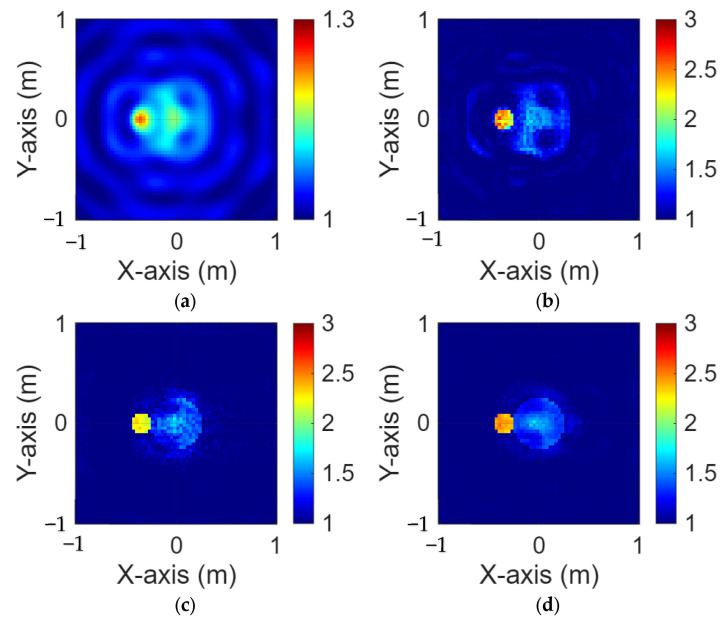
Reconstruction results of “FoamDielExt” database for $\varepsilon_{z}$. (**a**) BPS initial guess. (**b**) U-Net reconstruction image. (**c**) PGAN reconstruction image. (**d**) PGAN with SA reconstruction image.

**Figure 11:**
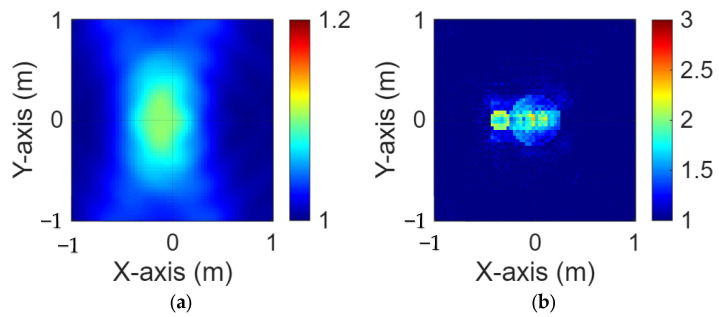

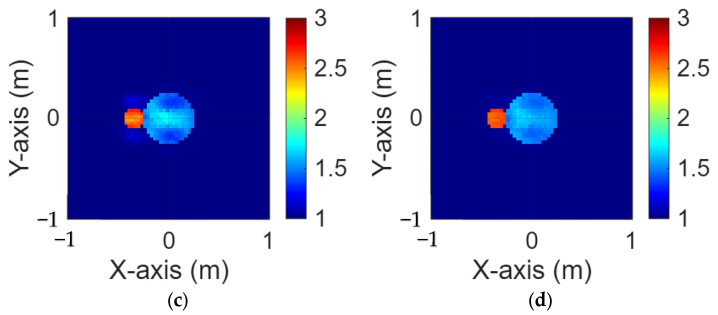
Reconstruction results of “FoamDielExt” database for $\varepsilon_{x}$. (**a**) BPS initial guess. (**b**) U-Net reconstruction image. (**c**) PGAN reconstruction image. (**d**) PGAN with SA reconstruction image.

**Figure 12:**
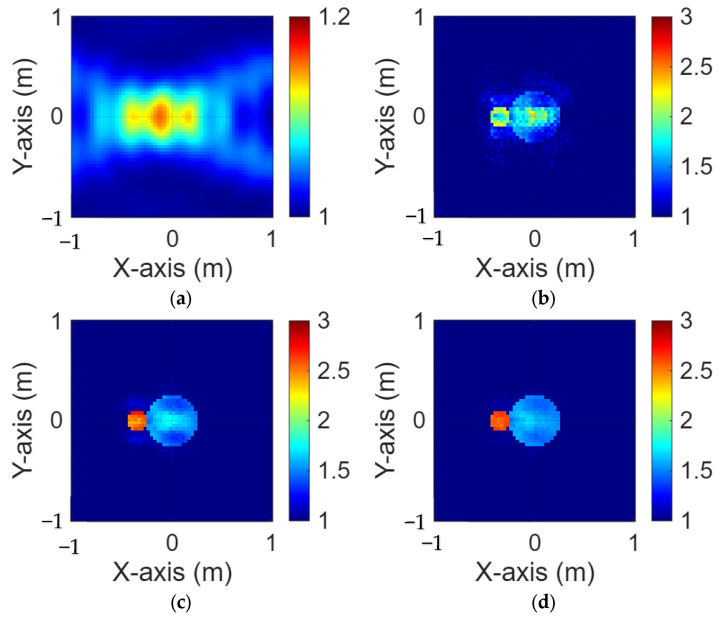
Reconstruction results of “FoamDielExt” database for $\varepsilon_{y}$. (**a**) BPS initial guess (**b**) U-Net reconstruction image (**c**) PGAN reconstruction image (**d**) PGAN with SA reconstruction image.

Figure 10, Figure 11 and Figure 12 show the reconstruction results of the “FoamDielExt” database for $\varepsilon_{z}$, $\varepsilon_{x}$, and $\varepsilon_{y}$, respectively. The BPS initial guesses are also presented to show the input information used by the learning-based reconstruction models. Compared with the BPS initial guesses, U-Net, PGAN, and PGAN with SA all provide improved dielectric profiles. However, the U-Net results are relatively smooth and contain less distinct structural details. PGAN further improves the reconstruction quality by introducing perceptual and adversarial learning. Compared with U-Net and PGAN, PGAN with SA provides clearer object boundaries and more accurate dielectric distributions, indicating that the self-attention mechanism is beneficial for experimental anisotropic dielectric reconstruction. The quantitative results listed in Table 5 further confirm the advantage of PGAN with SA. For $\varepsilon_{z}$, the RMSE decreases from 11.55% for U-Net and 8.24% for PGAN to 6.54% for PGAN with SA, while the SSIM increases from 50.34% and 84.13% to 87.76%, respectively. For $\varepsilon_{x}$, PGAN with SA achieves the lowest RMSE of 3.79% and the highest SSIM of 97.26%. Similarly, for $\varepsilon_{y}$, PGAN with SA reduces the RMSE to 3.80% and improves the SSIM to 97.18%. These results demonstrate that PGAN with SA improved reconstruction accuracy and structural similarity compared with both U-Net and the standalone PGAN for the experimental “FoamDielExt” database.

In this study, a perceptual generative adversarial network (PGAN) with an embedded self-attention (SA) mechanism was proposed to address the anisotropic electromagnetic inverse scattering problem. By integrating perceptual loss, self-attention, and SSIM-guided optimization, the proposed framework is able to better preserve structural and semantic consistency between the reconstructed and ground-truth dielectric profiles, while improving the recovery of edges, textures, and fine-scale features in anisotropic media. To evaluate the computational complexity of the proposed method, PGAN and PGAN with SA were compared in terms of training time, GPU memory usage, inference time, and the number of learnable parameters. PGAN required approximately 2 min for training and 5.06 GB of CPU memory and 6.6 GB of GPU memory, whereas PGAN with SA required approximately 2 min 10 s for training and 5.08 GB of CPU memory and 6.8 GB of GPU memory. The inference time of both models was less than 1 s per reconstruction. In terms of model size, PGAN contained approximately 7.9 million learnable parameters, while PGAN with SA contained approximately 8.1 million learnable parameters. These results indicate that the self-attention mechanism introduces only a small computational overhead while improving reconstruction accuracy and structural similarity.

## 5. Conclusions

In addition, this work considered the reconstruction of anisotropic objects based on BPS-generated initial estimates, with the formulation further adapted to account for the inherent complexity of anisotropic media. Compared with the TM case, the TE polarization is more challenging because the coupling between the x- and y-directed field components gives rise to stronger multiple scattering effects and more complicated electromagnetic interactions, making the corresponding inverse scattering problem more ill-posed. Nevertheless, the proposed framework remained effective for reconstructing the anisotropic permittivity components under both TM and TE cases. The feasibility and effectiveness of the proposed method were validated through extensive numerical and experimental studies, including homogeneous objects, inhomogeneous dielectric targets, MNIST-based dielectric patterns, and the FoamDielExt experimental dataset. Both qualitative and quantitative results consistently showed that PGAN with SA outperforms the original PGAN in terms of RMSE, SSIM, boundary preservation, and structural fidelity. Overall, these results confirm that the proposed framework provides a reliable and effective approach for high-resolution reconstruction of anisotropic dielectric distributions under various scattering conditions. Although the proposed PGAN with SA improves reconstruction accuracy under the tested noise conditions, its reconstruction capability may still be limited when the noise level becomes excessively high or when the measured data contain severe calibration errors, modeling mismatches, or forward-model mismatch. In practical scenarios, such mismatch may arise from different mesh resolutions, frequency shifts, antenna position errors, or uncertainties in the dielectric properties. Future work will focus on extending the present approach to more challenging experimental scenarios, stronger noise conditions, mismatched forward-model settings, and more advanced generative architectures to further improve robustness and reconstruction accuracy.

## Figures and Tables

**Table 1:** Performance of homogeneous dielectric object with permittivity between 1 and 1.5 under 20% Gaussian noise.

Performance		PGAN	PGAN with SA
$\varepsilon_{z}$	RMSE	4.45%	4.28%
	SSIM	91.19%	91.63%
$\varepsilon_{x}$	RMSE	5.06%	4.72%
	SSIM	78.47%	80.84%
$\varepsilon_{y}$	RMSE	6.54%	6.12%
	SSIM	76.45%	79.59%

**Table 2:** Performance of inhomogeneous dielectric object with permittivity between 1.5 and 2 under 5% Gaussian noise.

Performance		PGAN	PGAN with SA
$\varepsilon_{z}$	RMSE	8.46%	7.93%
	SSIM	88.7%	89.21%
$\varepsilon_{x}$	RMSE	6.63%	5.08%
	SSIM	92.13%	95.6%
$\varepsilon_{y}$	RMSE	7.25%	5.68%
	SSIM	92.08%	95.59%

**Table 3:** Performance of MNIST with permittivity between 2 and 2.5 under 5% Gaussian noise.

Performance		PGAN	PGAN with SA
$\varepsilon_{z}$	RMSE	10.77%	10.72%
	SSIM	86.59%	86.96%
$\varepsilon_{x}$	RMSE	11.85%	10.85%
	SSIM	83.9%	88%
$\varepsilon_{y}$	RMSE	12.5%	11.3%
	SSIM	84.02%	87.83%

**Table 4:** Quantitative performance comparison of different loss function.

Performance		$L_{1}$	$L_{1}+\beta L_{A}$	$L_{1}+\beta L_{A}+\gamma L_{PA}$	Our Method
$\varepsilon_{z}$	RMSE	12.12%	11.26%	10.87%	10.59%
	SSIM	83.52%	84.62%	85.65%	86.94%
$\varepsilon_{x}$	RMSE	11.23%	11.01%	10.01%	9.92%
	SSIM	85.22%	85.46%	90.43%	90.67%
$\varepsilon_{y}$	RMSE	11.84%	11.38%	10.67%	10.43%
	SSIM	85.72%	86.01%	90.22%	90.36%

**Table 5:** Quantitative performance comparison on the “FoamDielExt” database.

Performance		U-Net	PGAN	PGAN with SA
$\varepsilon_{z}$	RMSE	11.55%	8.24%	6.54%
	SSIM	50.34%	84.13%	87.76%
$\varepsilon_{x}$	RMSE	10.74%	4.68%	3.79%
	SSIM	81.26%	95.13%	97.26%
$\varepsilon_{y}$	RMSE	10.71%	4.82%	3.8%
	SSIM	80.57%	95.97%	97.18%

## Data Availability

The original contributions presented in the study are available on request from the corresponding author.
